# Diagnoses of Mental Health Disorders Among U.S. Active Component Service Members, 2020–2024

**Published:** 2026-02-04

## Abstract

Mental health disorders have long been recognized as a problem in a wide range of domains, including the military, resulting in significant impacts on general morbidity, health care provision, disability, and military discharges. From 2020 through 2024, a total of 560,035 U.S. active component service members were diagnosed with at least 1 mental health disorder. Annual incidence rates of mental health disorder increased steadily from 2020 until 2022, but adjustment disorder decreased since then, anxiety gradually increased, and the remaining conditions remained relatively unchanged. Most mental health disorder diagnoses were attributable to adjustment disorders, anxiety disorders, depressive disorders, post-traumatic stress disorder, alcohol-related disorder, and other mental health disorders. Historically, mental health disorders have often been misunderstood and stigmatized, leading to under-reporting, delayed treatment, and poor prognoses. Reflecting the unique stressors and cultural stigmas of military life, ongoing efforts to raise awareness, encourage help-seeking, and improve treatment options are essential to supporting the mental and emotional well-being of service members.

**What are the new findings?**

While the incidence of U.S. service members who were diagnosed with at least 1 mental health disorder remained stable from 2023 to 2024, the annual incidence rate of anxiety disorders demonstrated a continual increase from 2020 to 2024.

**What is the impact on readiness and force health protection?**

The sustained incidence of mental health disorders (11,534.1 per 100,000 person-years) diagnosed among U.S. active component service members in addition to significant variations in relation to sex, service branch, occupation, and length of military service, underscores the need for targeted interventions along with continued monitoring to ensure force readiness.

Mental health is a significant public health issue for the U.S. military due to the unique stressors experienced by service members. Military service, especially deployment, is linked to higher rates of mental health issues both during and after service. While combat and deployments are major risk factors, even general military service can lead to mental health challenges. Mental health issues can manifest at any time but are particularly prevalent when individuals are in close proximity to combat situations or during the transition from active duty to civilian life. ^1^

In 2024, mental health disorders accounted for the largest total number of hospital bed days and second highest total number of medical encounters for members of the active component of the U.S. Armed Forces. ^2^ In general, incidence rates (IRs) of mental health disorders have been observed to be highest among Army soldiers, female service members, and those in younger age groups. ^3 - 6^ The most recent *MSMR* update on mental health disorders, in 2024, found the IR of any mental health diagnosis increased by almost 40% between 2019 and 2023, largely attributable to adjustment disorders, anxiety disorders, depressive disorders, post-traumatic stress disorder (PTSD), alcohol-related disorders, as well as ‘other’ mental health disorders. ^6^ Mental health disorders often co-occur with other conditions, making professional diagnosis and personalized treatment plans crucial.

Despite the high prevalence and severity of mental health issues during military service, service members face challenges in accessing mental health treatment due to constraints including deployment, frequent relocation, limited mental health service capacity, and stigma associated with seeking care. ^7^ Addressing mental health disorders in military service members necessitates increased awareness, expanded access to care, and a prioritized focus on evidence-based treatments. Due to the significant impacts of mental health issues, military leaders, policy-makers, researchers, and the public are urging governments to provide timely and appropriate mental health services to service members. ^1^

This report summarizes the numbers, types, and IRs of mental health disorder diagnoses among U.S. active component service members (ACSMs) over a 5-year surveillance period, 2020 through 2024.

## Methods

The surveillance period for this report included January 1, 2020 through December 31, 2024. The surveillance population included all individuals who served in the active components of the U.S. Army, Navy, Air Force, Marine Corps, Coast Guard, or Space Force, at any time during the surveillance period. Due to Space Force personnel data availability for 2023 only, Space Force service members were combined with Air Force personnel for this analysis.

All data used to determine mental health diagnoses were derived from records routinely maintained in the Defense Medical Surveillance System (DMSS). DMSS records document both ambulatory health care encounters and hospitalizations of active component members of the U.S. Armed Forces in fixed military and civilian (if reimbursed through the Military Health System, or MHS) hospitals and clinics. Diagnoses were also derived from records of medical encounters of deployed service members documented in the Theater Medical Data Store (TMDS) in DMSS.

For purposes of analysis, mental health disorders were ascertained from records of medical encounters that included mental health disorder-specific diagnoses with International Classification of Diseases, 9th and 10th revisions (ICD-9 / ICD-10) codes (ICD-9: 290–319; ICD-10: F01–F99) **(Table 1)** in the first or second diagnostic position. Although the MHS transitioned to ICD-10 coding on October 1, 2015, ICD-9 codes were included in this analysis, as some TMDS encounters still contain ICD-9 diagnoses, which were needed to identify and exclude prevalent cases in records before October 1, 2015. Diagnoses of pervasive developmental disorder (ICD-9: 299.*; ICD-10: F84.*), specific delays in development (ICD-9: 315.*; ICD-10: F80.*– F82.*, F88–F89), mental retardation (ICD-9: 317.*–319.*; ICD-10: F70–F79), tobacco use disorder and nicotine dependence (ICD-9: 305.1; ICD-10: F17.*), and post-concussion syndrome (ICD-9: 310.2; ICD-10: F07.81) were excluded from analysis.

**TABLE 1.** Mental Health Disorder Classifications

Category	ICD–9 Codes	ICD–10 Codes
Acute stress disorders	308 *	F43.0
Adjustment disorders	309.0, 309.1, 309.2, 309.21, 309.22, 309.23, 309.24, 309.28, 309.29, 309.3, 309.4, 309.8, 309.82, 309.83, 309.89, 309.9	F43.2 * , F43.8 * , F43.9, F93.0, F94.8, F94.9
Alcohol-related disorders	291.0, 291.81, 303.0, 303.01, 303.02, 303.03, 303.9, 303.91, 303.92, 303.93, 305.0, 305.01, 305.02, 305.03	F10.1 * , F10.2 *
Substance-related disorders	304 * , 305.2 * –305.9 *	F11.1 * , F11.2 * , F121 * , F12.2 * , F13.1 * , F13.2 * , F14.1 * , F14.2 * , F15.1 * , F15.2 * , F16.1 * , F16.2 * , F18.1 * , F18.2 * , F19.1 * , F19.2 *
Anxiety disorders	300.0 * , 300.2 * , 300.3	F40 * , F41 * , F42 *
Post-traumatic stress disorder	309.81	F43.1 *
Depressive disorders	296.2, 296.21, 296.22, 296.23, 296.24, 296.25, 296.26, 296.3, 296.31, 296.32, 296.33, 296.34, 296.35, 296.36, 296.82, 296.9, 296.99, 300.4, 311.0	F32 * , F33 * , F34, F34.1, F34.8, F34.81, F34.89, F34.9, F39,
Eating disorders	307.1, 307.5, 307.51, 307.59	F50.0 * , F50.2 * , F50.8 * , F50.9
Factitious disorders	300.16, 300.19, 301.51	F68.1 *
Bipolar disorder	296.0, 296.01, 296.02, 296.03, 296.04, 296.05, 296.06, 296.1, 296.11, 296.12, 296.13, 296.14, 296.15, 296.16, 296.4, 296.41, 296.42, 296.43, 296.44, 296.45, 296.46, 296.5, 296.51, 296.52, 296.53, 296.54, 296.55, 296.56, 296.6, 296.61, 296.62, 296.63, 296.64, 296.65, 296.66, 296.7, 296.8, 296.81, 296.89, 301.13	F30 * , F31 * , F34.0
Personality disorders	301.0, 301.1, 301.11, 301.12, 301.2, 301.21, 301.22, 301.3, 301.4, 301.5, 301.59, 301.6, 301.7, 301.8, 301.81, 301.82, 301.83, 301.84, 301.89, 301.9	F21, F60 *
Schizophrenia	295 *	F20 * , F25 *
Psychotic disorders (other psychoses)	293.81, 293.82, 297.0, 297.1, 297.2, 297.3, 297.8, 297.9, 298.0, 298.1, 298.2, 298.3, 298.4, 298.8, 298.9	F06.0, F06.2, F22, F23, F24, F28, F29
Other mental health diagnoses	Any other not excluded code 290–319	Any other not excluded code F01–F99
Exclusions	299 * , 305.1, 310.2, 315 * , 317 * –319 *	F17 * , F0781, F70–F79, F80 * , F81 * , F82 * , F84 * , F88–F89

Abbreviations: ICD-9, International Classification of Diseases, 9th Revision; ICD-10, International Classification of Diseases, 10th Revision; PTSD, post-traumatic stress disorder

* Asterisk (*) indicates that any subsequent digit, character included.

Each incident diagnosis of a mental health disorder was defined using the corresponding Armed Forces Health Surveillance Case Definition. ^5^ For most mental health disorders, a case was defined by either a hospitalization with an indicator diagnosis in the first or second diagnostic position; 2 outpatient or TMDS visits within 180 days documented with indicator diagnoses (from the same mental health disorder category) in the first or second diagnostic position; or a single outpatient visit in a psychiatric or mental health care specialty setting (defined by Medical Expense and Performance Reporting System [MEPRS] code beginning with ‘BF’) with an indicator diagnosis in the first or second diagnostic position.

The surveillance case definitions for schizophrenia, acute stress disorder, and eating disorders included some exceptions to the case parameters described. The case definition for schizophrenia required either a single hospitalization with a diagnosis of schizophrenia in the first or second diagnostic position or 4 outpatient or TMDS encounters with a diagnosis of schizophrenia in the first or second diagnostic position. Schizophrenia cases who remained in the military for more than 2 years after becoming incident cases were excluded, as those cases were assumed to have been mis-diagnosed. The case definition for acute stress disorders required 1 encounter with an indicator diagnosis in any diagnostic position, due to the transient nature of its symptoms. Eating disorder cases required 1 inpatient encounter with an indicator diagnosis in the first or second diagnostic position, or a single outpatient or TMDS encounter with an indicator diagnosis in the primary diagnostic position.

Service members diagnosed with 1 or more mental health disorders before the surveillance period (i.e., prevalent cases) were not considered at risk of incident diagnoses of the same conditions during the period. Service members diagnosed with more than 1 mental health disorder during the surveillance period were considered incident cases in each category in which they fulfilled the case-defining criteria. Service members could be considered incident cases only once in each specific mental health disorder category.

## Results

### Numbers and incidence rates of mental health diagnoses

During the 5-year surveillance period, 560,035 ACSMs were diagnosed with at least 1 mental health disorder; of those individuals, 268,480 (47.9%) were diagnosed with mental health disorders in more than 1 diagnostic category **(Table 2)** . Overall, 1,007,037 incident diagnoses of mental health disorders were recorded in all diagnostic categories. The annual IRs of at least 1 mental health disorder increased from 8,430.2 per 100,000 person-years (p-yrs) in 2020 to 11,679.0 per 100,000 p-yrs in 2023, then decreased slightly to 11,534.1 per 100,000 p-yrs in 2024 **(Table 2)** .

**TABLE 2.** Incident Diagnoses and Rates of Mental Health Disorders, Active Component, U.S. Armed Forces, 2020–2024

		Total, 2020–2024		2020		2021		2022		2023		2024	
Category ^a^	No.	Rate ^b^	No.	Rate ^b^	No.	Rate ^b^	No.	Rate ^b^	No.	Rate ^b^	No.	Rate ^b^
Adjustment disorders	282,883	5,000.3	50,521	4,289.0	61,158	5,203.9	62,204	5,513.5	56,051	5,116.7	52,949	4,900.6
Anxiety disorders	208,217	3,467.3	28,237	2,254.8	37,651	3,007.7	45,351	3,768.5	48,685	4,190.6	48,293	4,251.6
Depressive disorders	177,483	2,914.0	26,839	2,129.4	34,455	2,728.0	39,117	3,209.1	39,439	3,331.3	37,633	3,231.9
Other mental health disorders	124,142	2,030.1	20,197	1,604.9	24,357	1,926.4	26,748	2,183.7	26,019	2,181.1	26,821	2,284.1
PTSD	95,189	1,491.3	13,062	993.9	17,310	1,312.1	20,835	1,630.2	22,385	1,797.6	21,597	1,761.6
Alcohol-related disorders	69,248	1,093.4	13,214	1,019.0	14,245	1,091.7	14,886	1,174.1	13,589	1,096.0	13,314	1,087.8
Personality disorders	15,668	239.0	2,772	206.6	3,296	244.2	3,585	273.1	3,186	248.1	2,829	223.3
Substance-related disorders	15,275	232.8	2,971	221.3	3,361	248.8	3,488	265.5	2,914	226.6	2,541	200.3
Bipolar disorder	8,654	131.7	1,493	111.0	1,833	135.4	1,961	149.0	1,806	140.2	1,561	122.9
Other psychoses	3,838	58.3	734	54.5	845	62.4	820	62.2	765	59.3	674	53.0
Eating disorders	3,678	55.9	502	37.3	708	52.3	885	67.2	808	62.7	775	61.0
Schizophrenia	1,475	22.4	261	19.4	310	22.9	309	23.4	314	24.3	281	22.1
Acute stress disorder	1,191	18.1	201	14.9	262	19.3	291	22.1	221	17.1	216	17.0
Factitious disorders	96	1.5	17	1.3	16	1.2	24	1.8	18	1.4	21	1.7
Total	1,007,037		161,021		199,807		220,504		216,200		209,505	
Individuals, *n*												
>1 type of diagnosis ^c^	268,480	4,074.7	33,831	2,509.5	43,445	3,202.8	48,856	3,702.7	47,853	3,706.1	45,954	3,608.0
Any diagnosis ^d^	560,035	8,499.7	113,651	8,430.2	139,110	10,255.4	152,615	11,566.5	150,798	11,679.0	146,907	11,534.1

Abbreviations: No., number; PTSD, post-traumatic stress disorder; *n* , number.

a An individual may be a case within a category only once per lifetime (censored person-time).

b Rate per 100,000 person-years.

c Rate per 100,000 person-years (individual continually at risk- uncensored person-time).

d Defined as unique occurrence of any mental health diagnosis.

Over the entire surveillance period, 95% of all incident mental health disorder diagnoses were attributable to adjustment disorders (n=282,883, 28.1%), anxiety disorders (n=208,217, 20.7%), depression disorders (n=177,483, 17.6%), ‘other’ mental health disorders (n=124,142, 12.3%), PTSD (n=95,189, 9.5%), and alcohol-related disorders (n=69,248, 6.9%) **(Table 2)** . In comparison, a relatively small number of incident diagnoses of personality disorders (n=15,668, 1.6%), substance-related disorders (n=15,275, 1.5%), bipolar disorder (n=8,654, 0.9%), other psychoses (n=3,838, 0.4%), eating disorders (n=3,678, 0.4%), schizophrenia (n=1,475, 0.1%), acute stress disorders, (n=1,191, 0.1%), and factitious disorders (n=96, 0.01%) contributed to the incident diagnoses of mental health disorders among ACSMs.

Annual IRs for adjustment disorders, alcohol-related disorder, personality disorders, substance-related disorder, bipolar disorder, eating disorders, and acute stress disorder increased steadily from 2020 until 2022 but then decreased, with adjustment disorders decreasing considerably thereafter. In contrast, anxiety increased gradually and steadily over the 5-year surveillance period, while conditions including depression, other mental health disorders, PTSD, other psychoses, and schizophrenia fluctuated **(Table 2)** .

### Co-occurring mental health diagnoses

Individuals with mental health disorders are often diagnosed with more than 1 mental health disorder. During the surveillance period, adjustment disorders were often co-diagnosed with other disorders, with 35.7% of substance-related disorders and 59.8% of personality disorders co-diagnosed with adjustment disorders. Depressive disorders were also often codiagnosed with all other mental health disorders, ranging from 26.7% of substance-related disorder cases with codiagnoses to 59.9% of bipolar disorder diagnoses. Incident cases of anxiety disorders were also co-diagnosed with factitious disorders (51.0%), bipolar disorder (47.3%), depressive disorders (46.0%), eating disorders (44.8%), PTSD (41.7%), personality disorders (41.3%), and acute stress disorder (36.4%) **(Table 3)** .

**TABLE 3.** Co-Morbid Incident Mental Health Disorder Diagnoses ^a^ , Active Component, U.S. Armed Forces, 2020–2024

		Adjustment		Alcohol-related		Substance-related		Anxiety		PTSD		Depression		Bipolar	
	No.	%	No.	%	No.	%	No.	%	No.	%	No.	%	No.	%
Adjustment disorders	282,883	—	26,174	37.8	5,451	35.7	87,606	42.1	42,762	44.9	86,450	48.7	3,871	44.7
Alcohol-related disorders	26,174	9.3	69,248	—	7,195	47.1	17,501	8.4	9,682	10.2	21,464	12.1	1,531	17.7
Substance-related disorders	5,451	1.9	7,195	10.4	15,275	—	3,153	1.5	1,500	1.6	4,084	2.3	537	6.2
Anxiety disorders	87,606	31.0	17,501	25.3	3,153	20.6	208,217	—	39,733	41.7	81,610	46.0	4,097	47.3
PTSD	42,762	15.1	9,682	14.0	1,500	9.8	39,733	19.1	95,189	—	38,179	21.5	2,630	30.4
Depressive disorders	86,450	30.6	21,464	31.0	4,084	26.7	81,610	39.2	38,179	40.1	177,483	—	5,180	59.9
Bipolar disorder	3,871	1.4	1,531	2.2	537	3.5	4,097	2.0	2,630	2.8	5,180	2.9	8,654	—
Personality disorders	9,362	3.3	3,070	4.4	738	4.8	6,476	3.1	3,796	4.0	8,458	4.8	1,286	14.9
Schizophrenia	628	0.2	256	0.4	154	1.0	524	0.3	256	0.3	781	0.4	314	3.6
Other psychoses	1,791	0.6	723	1.0	475	3.1	1,346	0.7	675	0.7	1,896	1.1	767	8.9
Acute stress disorder	513	0.2	102	0.2	23	0.2	434	0.2	296	0.3	383	0.2	41	0.5
Eating disorders	1,663	0.6	440	0.6	67	0.4	1,648	0.8	999	1.1	1,752	1.0	169	2.0
Factitious disorders	57	0.0	10	0.0	3	0.0	49	0.0	14	0.0	46	0.0	7	0.1
Other mental health disorders	44,793	15.8	18,076	26.1	5,677	37.2	36,229	17.4	18,271	19.2	32,684	18.4	2,068	23.9
Total	282,883		69,248		15,275		208,217		95,189		177,483		8,654	

		Personality		Schizophrenia		Other		Acute Stress		Eating		Factitious		Other	
	No.	%	No.	%	No.	%	No.	%	No.	%	No.	%	No.	%
Adjustment disorders	9,362	59.8	628	42.6	1,791	46.7	513	43.1	1,663	45.2	57	59.4	44,793	36.1
Alcohol-related disorders	3,070	19.6	256	17.4	723	18.8	102	8.6	440	12.0	10	10.4	18,076	14.6
Substance-related disorders	738	4.7	154	10.4	475	12.4	23	1.9	67	1.8	3	3.1	5,677	4.6
Anxiety disorders	6,476	41.3	524	35.5	1,346	35.1	434	36.4	1,648	44.8	49	51.0	36,229	29.2
PTSD	3,796	24.2	256	17.4	675	17.6	296	24.9	999	27.2	14	14.6	18,271	14.7
Depressive disorders	8,458	54.0	781	53.0	1,896	49.4	383	32.2	1,752	47.6	46	47.9	32,684	26.3
Bipolar disorder	1,286	8.2	314	21.3	767	20.0	41	3.4	169	4.6	7	7.3	2,068	1.7
Personality disorders	15,668	—	202	13.7	564	14.7	52	4.4	381	10.4	19	19.8	4,069	3.3
Schizophrenia	202	1.3	1,475	—	955	24.9	8	0.7	17	0.5	4	4.2	443	0.4
Other psychoses	564	3.6	955	64.8	3,838	—	20	1.7	32	0.9	14	14.6	1,200	1.0
Acute stress disorder	52	0.3	8	0.5	20	0.5	1,191	—	8	0.2	0	0.0	343	0.3
Eating disorders	381	2.4	17	1.2	32	0.8	8	0.7	3,678	—	2	2.1	1,273	1.0
Factitious disorders	19	0.1	4	0.3	14	0.4	0	0.0	2	0.1	96	—	37	0.0
Other mental health disorders	4,069	26.0	443	30.0	1,200	31.3	343	28.8	1,273	34.6	37	38.5	124,142	—
Total	15,668		1,475		3,838		1,191		3,678		96		124,142	

Abbreviation: PTSD, post-traumatic stress disorder; No., number.

a Mental health disorder diagnoses at any time during surveillance period.

### Incidence rates of mental health diagnoses by sex

In general, most incident mental health disorder diagnoses were more prevalent among female service members, but alcohol- and substance-related disorders were more prevalent in male service members during the 5-year surveillance period. Schizophrenia was diagnosed at a higher rate in male service members in 2024 and 2020 **(Figures 1a** – **2b)** .

**FIGURE 1a.**
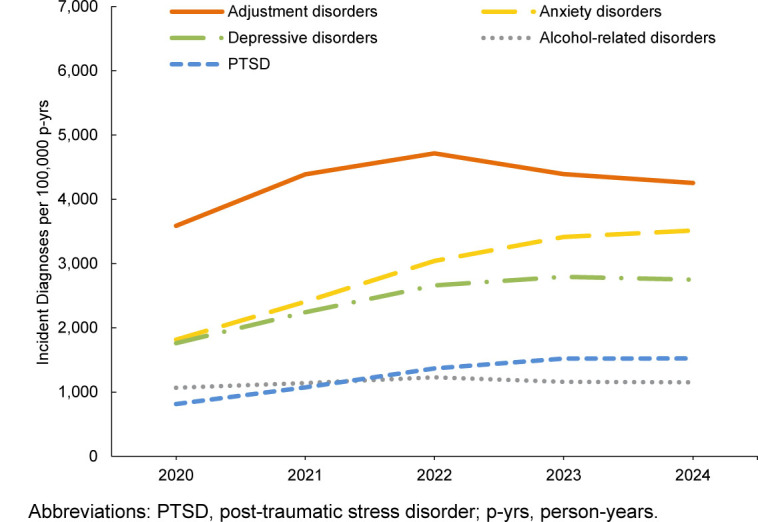
Annual Incidence Rates of the Leading 5 Mental Health Disorder Diagnoses, Active Component Men, U.S. Armed Forces, 2020–2024

**FIGURE 1b.**
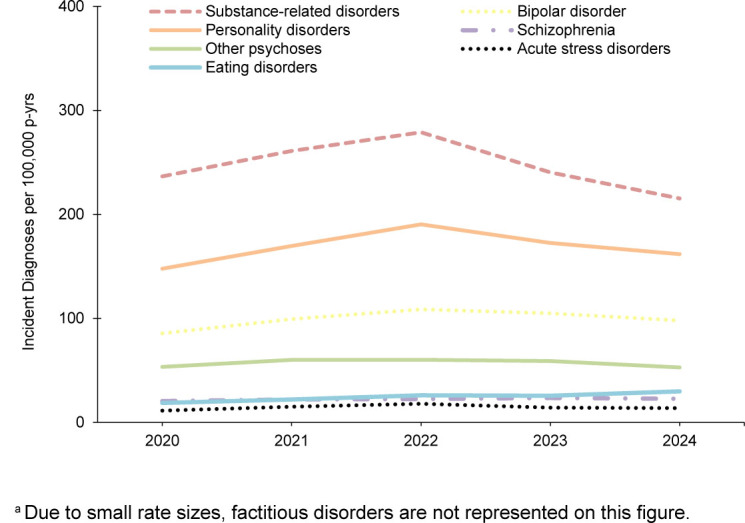
Annual Incidence Rates ^a^ of the Next Most Frequent Mental Health Disorder Diagnoses, Active Component Men, U.S. Armed Forces, 2020–2024

**FIGURE 2a.**
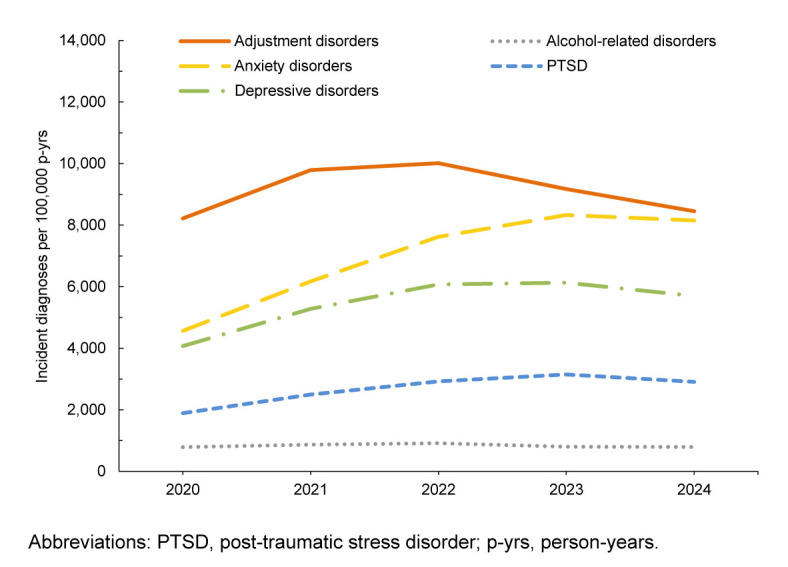
Annual Incidence Rates of the Leading 5 Mental Health Disorder Diagnoses, Active Component Women, U.S. Armed Forces, 2020–2024

**FIGURE 2b.**
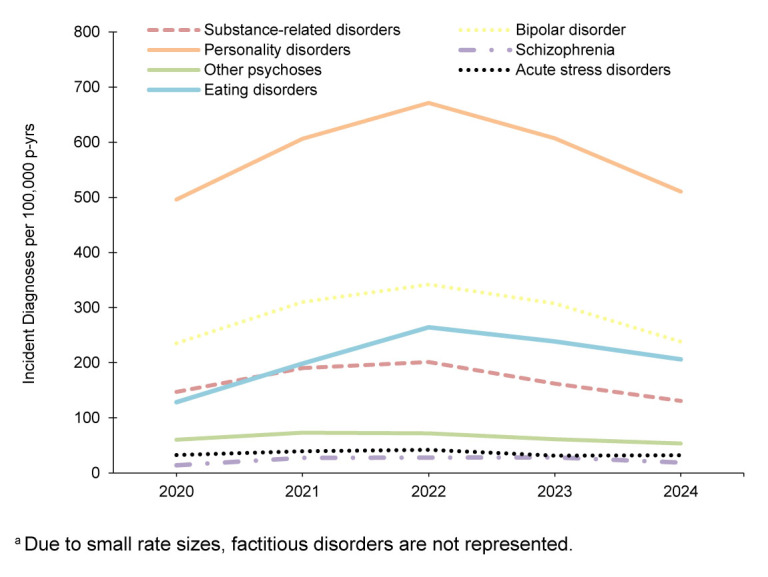
Annual Incidence Rates ^a^ of Next Most Frequent Mental Health Disorder Diagnoses, Active Component Women, U.S. Armed Forces, 2020–2024

Rates of mental health disorder diagnoses in male service members steadily increased until 2022, remaining relatively unchanged since then, with the exception of decreases in adjustment disorders, substance-related disorders, and personality disorders. Anxiety disorders increased throughout the surveillance period among male service members **(Figures 1a** , **1b)** .

Rates of mental health disorder diagnoses in female service members followed a similar pattern to those of male service members, with the exception of a slight decrease in anxiety disorders in 2024. Adjustment disorder IRs were the highest among women in 2024, followed by anxiety disorders, depressive disorders, other mental health disorders, and PTSD. During the 5-year surveillance period, eating disorders were 7–10 times more common in female ACSMs than in males, while personality disorders were 3.2–3.6 times more common among women **(Figures 2a** , **2b)** .

### Incidence rates of mental health diagnoses by age

Rates of most mental health disorders varied by age, with adjustment disorders exhibiting the highest incidence among all age groups **(Figure 3)** . Service members under age 20 years had the highest IR of adjustment disorder, compared to all other age groups. Rates of alcohol- and substance-related disorders, along with personality disorders, bipolar disorder, eating disorders, and schizophrenia, were highest for service members aged 20-24 years, while declining thereafter with increasing age. As age increased, PTSD increased, while adjustment disorders, anxiety disorders, depressive disorders, and acute stress disorders fluctuated. ACSMs ages 40-49-years had the highest IRs of anxiety and depressive disorders, and those older than age 50 years had the highest incidence of PTSD. After age 30 years, rates of adjustment disorders, anxiety disorders, and depressive disorders increased until ages 40-49 years, thereafter declining in those older than age 50 years.

**FIGURE 3.**
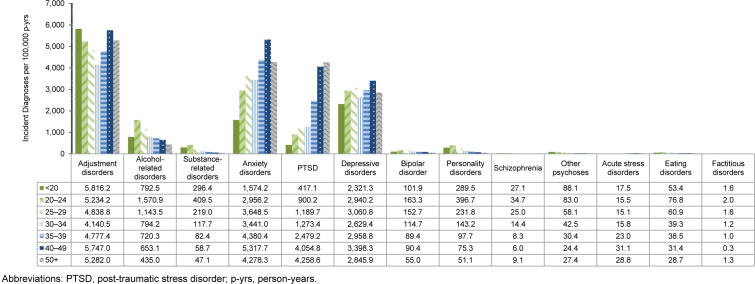
Incidence Rates of Mental Health Disorder Diagnoses by Category and Age Group, Active Component, U.S. Armed Forces, 2020–2024

### Incidence rates of mental health diagnoses by service

Overall, IRs of mental health disorders were highest in the Army, specifically adjustment disorders, alcohol-related disorders, substance related disorders, anxiety disorders, PTSD, schizophrenia, other psychoses, and eating disorders. The Navy accounted for the highest IRs of depressive disorders, personality disorders, and bipolar disorder, while the Coast Guard accounted for the highest IRs of acute stress disorders **(Figure 4)** .

**FIGURE 4.**
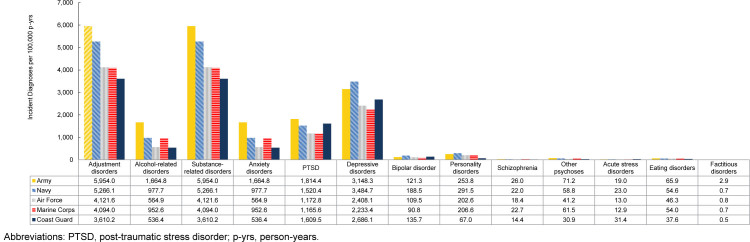
Incidence Rates of Mental Health Disorder Diagnoses by Category and Branch of Service, Active Component, U.S. Armed Forces, 2020–2024

### Incidence rates of mental health diagnoses by occupation

Rates of adjustment disorders, anxiety disorders, depressive disorders, PTSD, personality disorders, bipolar disorder, eating disorders, and acute stress disorders were generally highest in health care occupations. Service members in combat-related roles exhibited the highest IRs of alcohol- and substance-related disorders, while those in motor transport had the highest rates of other psychoses and schizophrenia. By contrast, pilots and air crew personnel showed the lowest IRs of mental health disorders **(Figure 5)** .

**FIGURE 5.**
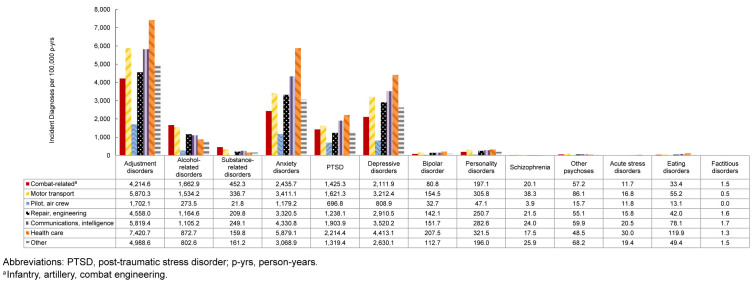
Incidence Rates of Mental Health Disorder Diagnoses by Category and Military Occupation, Active Component, U.S. Armed Forces, 2020–2024

### Incidence rates of mental health diagnoses by time in service

Rates of mental health disorder diagnoses differ by length of service, with highest IRs of schizophrenia, other psychoses, and acute stress disorders diagnoses occurring among ACSMs with less than 6 months of service. For those who served 12-36 months, the most common diagnoses were adjustment disorders, alcohol-related disorders, substance-related disorders, personality disorders, bipolar disorder, and eating disorders. Among those who served 36 months or longer, anxiety disorders, depressive disorders, and PTSD were most common **(Figure 6)** .

**FIGURE 6.**
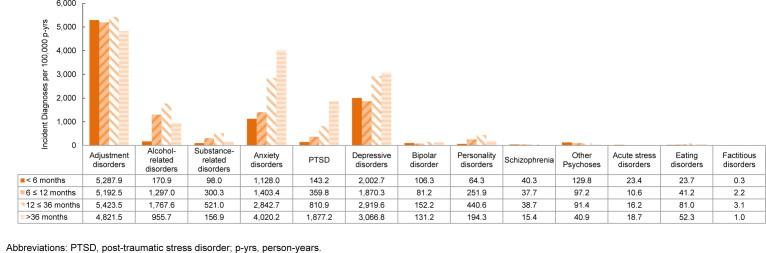
Incidence Rates of Mental Health Disorder Diagnoses by Category and Time in Service, Active Component, U.S. Armed Forces, 2020–2024

## Discussion

This report provides an update on incident diagnoses for mental health disorders among ACSMs of the U.S. Armed Forces from 2020 through 2024. Adjustment disorders, anxiety disorders, depressive disorders, PTSD, and alcohol-related disorder, along with other mental health disorders, consistently accounted for approximately 95% of all mental health disorder diagnoses during the 5-year surveillance period. IRs of anxiety disorders increased substantially from 2020 to 2024.

The increasing incidence of anxiety disorders and PTSD among ACSMs is complex, with multiple contributing factors including combat exposure, military culture and environment, personal and pre-existing factors, in addition to other stressors. ^8 , 9^ The consequences of these disorders can affect service readiness, military occupations, professional and personal relationships, long-term health, substance use, and potential suicidal ideation. ^10^

Prior *MSMR* reports indicate that approximately one-third of anxiety disorder diagnoses from 2000 to 2011 had a co-occurring diagnosis of either adjustment or depressive disorder. ^11^ Co-occurring diagnoses persist in this report, which documents both adjustment disorders (42.1%) and depressive disorders (39.2%) as the leading 2 co-occurring diagnoses, from 2020 through 2024, for ACSMs with incident anxiety disorder diagnoses. Co-occurring mental health diagnoses represent a significant challenge, as they can increase both the complexity and severity of symptomology, complicate diagnosis and treatment, and affect overall prognosis.

Mental health disorders affect male and female service members differently, with effects on both related prevalence and presentation of mental health conditions. During the 5-year surveillance period, most mental health disorders were more prevalent among female ACSMs, while alcohol and substance-related disorders were more common among male ACSMs. Female service members' vulnerability to physical and mental health issues appears to be highly correlated with unwanted gender-based experiences, which may lead them more likely to report mental health problems than male service members. ^12^ In particular, the IR of eating disorders among female service members in this report was 7–10 times higher than that of male service members, similar to the results of a previous report. ^13^ Eating disorders are complex conditions, difficult to treat and often co-occurring with other mental health conditions, making understanding each individual's unique needs and experiences crucial for effective treatment. ^14^ Differences in mental health disorder diagnoses between the sexes underscores the need for individualized treatment approaches that are sex-specific.

Consistent with previous findings, this report confirms age-related variations in mental health diagnoses, with service members aged 20-24 years exhibiting a particularly high incidence of mental health disorders during the 2020–2024 surveillance period. ^3 , 15^ While IRs varied by age group, each age group exhibited mental health problems that were particularly severe and unique to that age group.

From 2020 through 2024, the Army consistently reported higher IRs of most mental health disorders, likely due to its large size, frequent deployments, and high-stress missions. ^6 , 15^ While the Army has higher IRs overall, the Marine Corps is often viewed as the most mentally demanding branch due to its rigorous standards and intense emotional and psychological pressures. ^16^ Effective management and prevention must take into account each branch of service's distinct demographics, culture and missions, in order to fully address mental health.

As documented in a prior report, ^6^ service members in health care occupations exhibited higher rates of diagnoses of most mental health disorders. Health care professionals often struggle to provide appropriate care for themselves, and when mental illness develops, tend to be reluctant to seek help when needed and neglect self-care. ^17^ The higher rates of mental health disorders among those in health care occupations suggest an important need for future research on effective solutions to support the mental health of military health care personnel.

During the 5-year surveillance period, adjustment disorders generally had highest incidence rates during the early stages of military service. All mental health disorders continued to increase until mid-career, after which all mental health disorders decreased or remained stable through the later career stages, with the exception of anxiety disorders and PTSD.

There are several limitations in interpreting the results in this report. First, this report was compiled based on standardized administrative records and may not be reliable indicators of the true burden of mental health disorders among military service members. Second, this report may under-estimate the incidence of mental health disorders if service members do not seek appropriate care or receive care not routinely documented as ICD-9 / ICD-10-coded diagnoses (e.g., from private practitioners, counseling or advocacy support centers, chaplains), or if mental health disorders were not diagnosed or reported on standardized records of care, or if diagnoses were mis-coded or incorrectly transcribed on centrally transmitted records. Conversely, some conditions may have been erroneously diagnosed or mis-coded as mental health disorders (e.g., screening visits), which may contribute to an over-estimation of the true burden of disease. Lastly, these analyses summarize the experiences of individuals while serving in an active component of the U.S. military and do not include mental health disorders or problems that affected members of reserve components or veterans of recent military service who received care outside the MHS.

In September 2024, the Department of Defense revised Instruction 6490.08, establishing a Department policy that promotes health-seeking behaviors for mental health services. The new policy emphasizes unrestricted, non-stigmatizing access to mental health care services, including voluntary substance mis-use education, as essential for maintaining the health and readiness of the total force. ^18^ As the burden of mental health disorders continues to increase during a period of policy change, ongoing surveillance and further analyses are warranted to better understand the true burden of disease in addition to health care access and provision. The results from this report under-score the need for mental health services to address a range of mental health co-morbidities in ACSMs.

Mental health stigma is a primary barrier to help-seeking in the military, consistently identified as a major concern in military studies. ^19^ Although stigma's direct effect on care may be minimal, ^20^ a holistic approach that comprehensively addresses the complex needs of military personnel is crucial, providing integrated care from military and civilian providers that proactively works to reduce the persistent stigma associated with seeking mental health support. ^21 , 22^

The trends in this report demonstrate the ongoing need for mental health services among U.S. military members, documented in previous *MSMR* reports. Effectively addressing the increasing rates of anxiety disorders and PTSD in ACSMs requires evidence-informed prevention strategies, enhanced access to care, early intervention, appropriate and integrated treatment, strengthened support networks, along with ongoing research. ^1^ In addition, effective management of co-occurring disorders requires comprehensive assessment approaches complemented by treatment plans that are both individualized and integrated. ^8 , 23^

**FIGURE 3 SUPPLEMENT.**
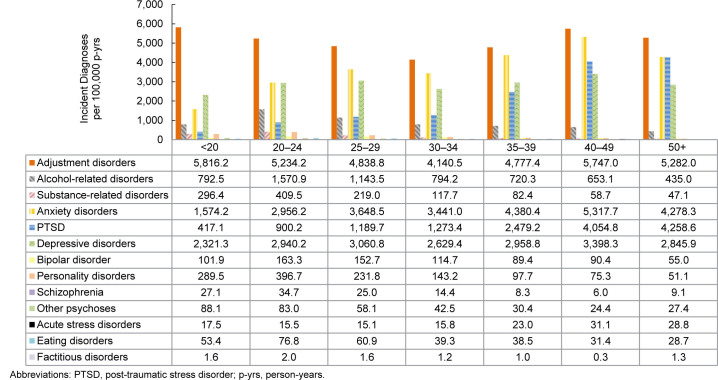
Incidence Rates of Mental Disorder Diagnoses by Age Group and Category, Active Component, U.S. Armed Forces, 2020–2024

**FIGURE 4 SUPPLEMENT.**
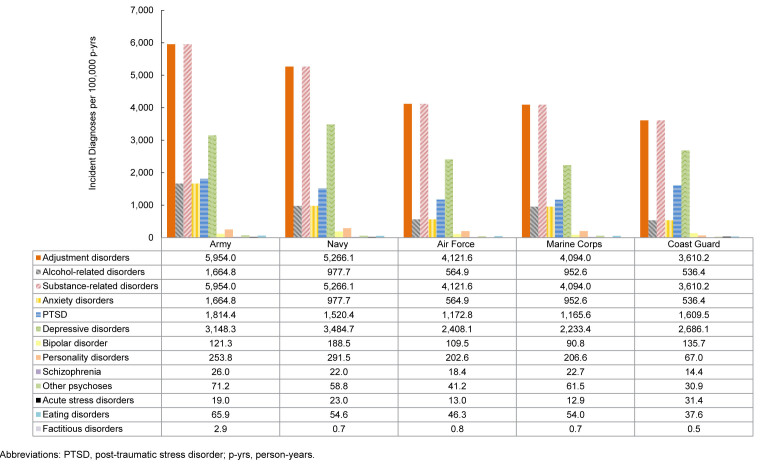
Incidence Rates of Mental Health Disorder Diagnoses by Branch of Service and Category, Active Component, U.S. Armed Forces, 2020–2024

**FIGURE 5 SUPPLEMENT.**
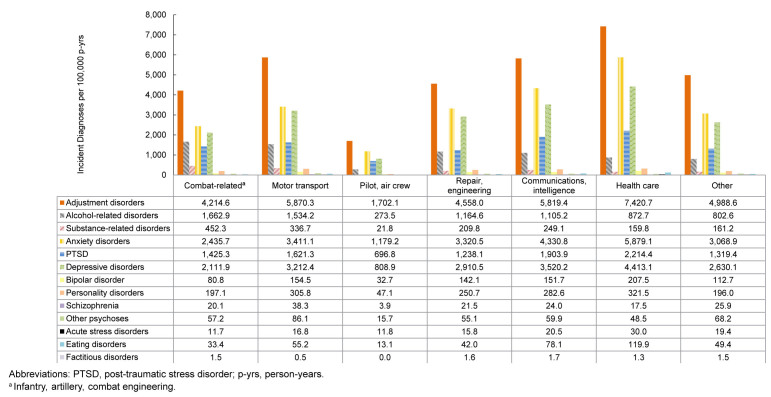
Incidence Rates of Mental Health Disorder Diagnoses by Military Occupation and Category, Active Component, U.S. Armed Forces, 2020–2024

**FIGURE 6 SUPPLEMENT.**
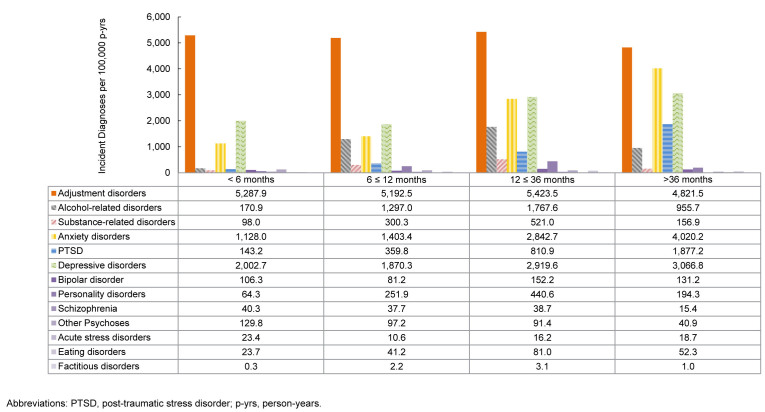
Incidence Rates of Mental Health Disorder Diagnoses by Time in Service and Category, Active Component, U.S. Armed Forces, 2020–2024
